# The Subcutaneous Adipose Microenvironment as a Determinant of Body Fat Development in Polycystic Ovary Syndrome

**DOI:** 10.1210/jendso/bvae162

**Published:** 2024-09-17

**Authors:** Daniel A Dumesic, Melody A Rasouli, Jessica D Katz, Gwyneth G Lu, Devyani Dharanipragada, Adina F Turcu, Tristan R Grogan, Kimberly E Flores, Clara E Magyar, David H Abbott, Gregorio D Chazenbalk

**Affiliations:** Department of Obstetrics and Gynecology, University of California, Los Angeles, Los Angeles, CA 90095, USA; Department of Obstetrics and Gynecology, University of California, Los Angeles, Los Angeles, CA 90095, USA; Department of Obstetrics and Gynecology, University of California, Los Angeles, Los Angeles, CA 90095, USA; Department of Obstetrics and Gynecology, University of California, Los Angeles, Los Angeles, CA 90095, USA; Department of Obstetrics and Gynecology, University of California, Los Angeles, Los Angeles, CA 90095, USA; Division of Metabolism, Endocrinology, Nutrition and Diabetes, University of Michigan, Ann Arbor, MI 48103, USA; Department of Medicine Statistics Core, University of California, Los Angeles, Los Angeles, CA 90024, USA; Department of Pathology and Laboratory Medicine, University of California, Los Angeles, Los Angeles, CA 90095, USA; Department of Pathology and Laboratory Medicine, University of California, Los Angeles, Los Angeles, CA 90095, USA; Department of Obstetrics and Gynecology, Wisconsin National Primate Research Center, University of Wisconsin, Madison, Madison, WI 53715, USA; Department of Obstetrics and Gynecology, University of California, Los Angeles, Los Angeles, CA 90095, USA

**Keywords:** adipose, polycystic ovary syndrome, androgen receptor, cortisol, aldo-keto reductase 1C3, activator protein-1

## Abstract

**Context:**

Adipose steroid metabolism modifies body fat development in polycystic ovary syndrome (PCOS).

**Objective:**

To determine whether subcutaneous (SC) abdominal adipose aldo-keto reductase 1C3 (AKR1C3; a marker of testosterone generation) is increased in normal-weight women with PCOS vs age- and body mass index (BMI)-matched normoandrogenic ovulatory women (controls) and is related to SC abdominal adipose activator protein-1 (AP-1; a marker of adipocyte differentiation) and/or androgen receptor (AR) protein expression in predicting fat accretion.

**Design:**

Prospective cohort study.

**Setting:**

Academic center.

**Patients:**

Eighteen normal-weight PCOS women; 17 age- and BMI-matched controls.

**Intervention(s):**

Circulating hormone/metabolic determinations, intravenous glucose tolerance testing, total body dual-energy x-ray absorptiometry, SC abdominal fat biopsy, immunohistochemistry.

**Main Outcome Measure(s):**

Clinical characteristics, hormonal concentrations, body fat distribution, SC adipose AKR1C3, AR, and AP-1 protein expression.

**Results:**

Women with PCOS had significantly higher serum androgen levels and greater android/gynoid fat mass ratios than controls. SC adipose AKR1C3, AR, and AP-1 protein expressions were comparable between the study groups, but groups differed in correlations. In PCOS women vs controls, SC adipose AKR1C3 protein expression correlated positively with android and gynoid fat masses and negatively with SC adipose AP-1 protein expression. SC adipose AR protein expression correlated negatively with fasting serum free fatty acid and high-density lipoprotein levels. In both study groups, SC adipose AKR1C3 protein expression negatively correlated with serum cortisol levels.

**Conclusion:**

In normal-weight PCOS women, SC abdominal adipose AKR1C3 protein expression, in combination with intra-adipose AP-1 and AR-dependent events, predicts fat accretion in the presence of physiological cortisol levels.

As a common endocrine-metabolic disorder of reproductive-aged women, polycystic ovary syndrome (PCOS) is characterized by ovarian hyperandrogenism from altered hypothalamic-pituitary-ovarian function in combination with preferential abdominal (android) fat accumulation worsened by obesity [1]. Most women with PCOS have insulin resistance from perturbed insulin receptor signaling, altered adipokine secretion, and abnormal steroid metabolism [1], which collectively promote glucose intolerance, dyslipidemia, and metabolic syndrome with increased adiposity [1, 2].

Adipose steroid metabolism can alter female body fat deposition through adipose-specific aldo-keto reductase 1C (AKR1C) enzymes [3-5]. As a major AKR1C isoform with 17β-hydroxysteroid dehydrogenase activity, AKR1C3 generates testosterone (T) from androstenedione (A4), with higher activity in subcutaneous (SC) than intra-abdominal fat [4, 6] accompanying enhanced SC fat storage in women [5]. Increased AKR1C3-mediated T generation in SC abdominal adipose tissue of overweight/obese women with PCOS is linked with decreased lipid mobilization (lipolysis) in vivo, while exposure of human adipocytes to androgens promotes lipid formation (lipogenesis) in vitro [7]. Furthermore, insulin-induced AKR1C3-mediated T generation in cultured human female SC adipocytes is blocked by the AKR1C3 inhibitor, 3-4-trifluoromethyl-phenylamino-benzoic acid, as is A4-induced lipogenesis, linking insulin-mediated AKR1C3 generation of T from A4 with enhanced SC fat storage [7].

In contrast, the serum T to A4 ratio, as an indirect enzymatic marker of AKR1C3 activity, is similar in normal-weight PCOS women compared to age- and body mass index (BMI)-matched normoandrogenic ovulatory women (controls), whereas an inverse relationship between serum T and cortisol levels instead predicts android fat mass in both study groups combined [8]. Nevertheless, in normal-weight PCOS women, *AKR1C3* overexpression accompanying exaggerated lipid accumulation in SC abdominal stem cells during adipocyte development in vitro [9-12] coincides with altered chromatin accessibility of activator protein-1 (AP-1) as a transcriptional regulator of cellular proliferation, differentiation, apoptosis, and inflammation [13].

Therefore, the primary hypothesis of the present paper is that SC abdominal adipose AKR1C3 protein expression is increased in normal-weight women with PCOS compared to age- and BMI-matched normoandrogenic ovulatory women (controls) and is related to SC abdominal adipose AP-1 and/or androgen receptor (AR) protein expression in predicting fat mass accretion. An additional exploratory hypothesis is that SC abdominal adipose AKR1C3 protein expression is related to serum cortisol level.

## Materials and Methods

### Study Participants

Eighteen normal-weight PCOS and 17 control women (19-35 years; 19-25 kg/m^2^) who had previously participated in our National Institutes of Health (NIH)-funded study (P50 HD071836) examining adipose function in PCOS [8-11, 14, 15] also had SC abdominal adipose tissue available for study. Previously generated data from this research protocol included measurements of circulating hormone/metabolite levels and body fat as well as distribution in normal-weight PCOS and control subjects [8-11, 14, 15], while the present paper addresses new data regarding AKR1C3, AR, and AP-1 protein expressions in SC abdominal adipose obtained from these same subjects. As published previously, each woman with PCOS was age-/BMI-matched to a normoandrogenic ovulatory (control) woman, who was similarly enrolled; all women were healthy individuals, although 1 PCOS subject had hypertriglyceridemia and a reduced insulin sensitivity index (Si) below the level of the general population [8-11, 14, 15].

PCOS was diagnosed by 1990 NIH criteria and biochemical hyperandrogenism, as previously defined by an elevated mean serum total or free T level from 2 separate blood samples >2 SD above the normal ranges of the age- and BMI- matched controls [8-11, 14, 15]. Control women had normal menstrual cycles at 21- to 35-day intervals and a luteal phase progesterone level without signs of androgen excess [1]. Exclusion criteria, including late-onset congenital adrenal hyperplasia, thyroid dysfunction, and hyperprolactinemia, have previously been reported [8-11, 14, 15]. All studies were performed according to the Declaration of Helsinki after approval by the University of California, Los Angeles Institutional Review Board and signed informed consent by each subject.

### Body Fat Distribution

Waist and hip measurements were determined in all subjects. Total body dual-energy x-ray absorptiometry scan was determined with a Hologic QDR Discovery A densitometer (Hologic, Inc., Bedford, MA), with android and gynoid fat regions measured from the first lumbar vertebra to the top of pelvis and from the femoral head to the mid-thigh, respectively, as previously reported [8-11, 14, 15]. During the COVID-19 pandemic, dual-energy x-ray absorptiometry scan also was measured with a Hologic Horizon A densitometer (Hologic, Inc.) in 3 PCOS women using the same mathematical software algorithms established by the manufacturer to partition soft tissue mass between its fat and lean components and to measure total, android, and gynoid fat masses by utilizing the same analytical methods.

### Blood Sampling

All blood sampling was performed during the follicular phase (days 5-10 of the menstrual cycle) in control women or during documented oligo-anovulation by a low serum follicular phase progesterone level in PCOS women, as previously reported [14]. Fasting blood samples were collected at 10 Am for adrenal and ovarian steroids: 11-hydroxyandrostenedione (11OHA4), as the most abundant adrenal 11-oxyandrogen [8, 16, 17], 17-hydroxyprogesterone, cortisol, cortisone, dehydroepiandrosterone sulfate (DHEAS), total and free T, A4, estrone (E1), estradiol (E2); and for gonadotropins, glucose, free fatty acid (FFA), insulin, SHBG, and lipids [total cholesterol, high-density lipoprotein cholesterol (HDL-C), low-density lipoprotein cholesterol (LDL-C), and triglyceride (TG)]. 11-hydroxyandrostenedione was chosen because it is quantitatively the dominant 11-oxygenated androgen of adrenal origin that serves as a substrate for other downstream metabolites [18-20] in women with and without PCOS [8, 16, 17].

Immediately thereafter, a frequently sampled intravenous glucose tolerance test was performed using the modified minimal model of Bergman [21], except in 1 control who declined the study. Briefly, glucose in 50% concentration (0.3 g/kg) and regular human insulin (0.03 units/kg) were injected intravenously under fasting conditions at 0 and 20 minutes, respectively, and blood was collected at −20, −15, −5, 0, 2, 4, 8, 19, 22, 30, 40, 50, 70, 90, and 180 minutes for glucose and insulin determinations. Mathematical modeling of circulating glucose and insulin levels defined Si (ie, insulin action to accelerate glucose uptake and suppress glucose production) and acute response to glucose (AIRg; ie, pancreatic β-cell response to glucose infusion).

### Hormonal and Metabolic Assays

Quantification of serum 11OHA4, cortisol, cortisone, T, and A4 was performed simultaneously by liquid chromatography-tandem mass spectrometry at the University of Michigan, Ann Arbor, as previously described [22, 23]. The intra-assay coefficient of variation (CVs) for all steroids were <3.5%, and all detection limits were >2.4 ng/dL.

Serum levels of DHEAS and E1 were measured by liquid chromatography-tandem mass spectrometry (Quest Diagnostics Nichols Institute, San Juan Capistrano, CA), as previously described [8-11, 14, 15]. The intra-assay CVs were DHEAS, 2.6% and E1, 10.2%. The interassay CVs were DHEAS, 4.4% and E1, 9.5%. The detection limits were DHEAS, 2 μg/dL and E1, 10 pg/mL. The Vermeulen equation was used to calculate free T from the concentrations of total T and SHBG [Beckman Coulter Cat# A48617, RRID:AB_2893035 (https://antibodyregistry.org/AB_2893035)], assuming a fixed albumin concentration of 4.3 g/dL [24].

Serum measurements of insulin (Roche Cat# 12017547, RRID:AB_2756877 [https://antibodyregistry.org/AB_2756877]), LH (Roche Cat# 11732234, RRID:AB_2800498 [https://antibodyregistry.org/AB_2800498]), FSH (Roche Cat# 11775863, RRID:AB_2800499 [https://antibodyregistry.org/AB_2800499]), and E2 (Roche, Cat# 03000079, RRID:AB_2893079 [https://antibodyregistry.org/AB_2893079]) by electrochemiluminescence, glucose by a hexokinase method, and fasting lipids by spectrophotometry were performed at the University of California, Los Angeles, Center for Pathology Research Services, as previously described [8-11, 14, 15]. The intra-assay CVs were insulin, 0.6%; LH, 1.0%; FSH, 2.1%; E2, 7.0%; glucose, 0.8%; total cholesterol, 0.7%; LDL-C, 0.5%; HDL-C, 0.6%; and TG, 0.6%. The interassays CVs were: insulin, 2.6%; LH, 2.3%; FSH, 2.8%; E2, 10.7%; glucose, 0.9%; total cholesterol, 1.0%; LDL-C, 1.2%; HDL-C, 0.9%; and TG, 0.7%. Detection limits were: insulin, <1 μU/mL; LH, <0.3 mIU/mL; FSH, <0.3 mIU/mL; E2, < 2 pg/mL; glucose, <10 mg/dL; total cholesterol, <11 mg/dL; LDL-C, <10 mg/dL; HDL-C, <4 mg/dL; and TG, <9 mg/dL

Serum FFAs were measured by quantitative spectrophotometry (ARUP Laboratories, Salt Lake City, UT). The intra- and interassay CVs for FFAs were 1.8% and 1.2%, respectively, as previously reported [8-11, 14, 15]. The detection limit for FFA was 0.01 mmol/L.

### Immunohistochemical Analysis

**Figure 1.**
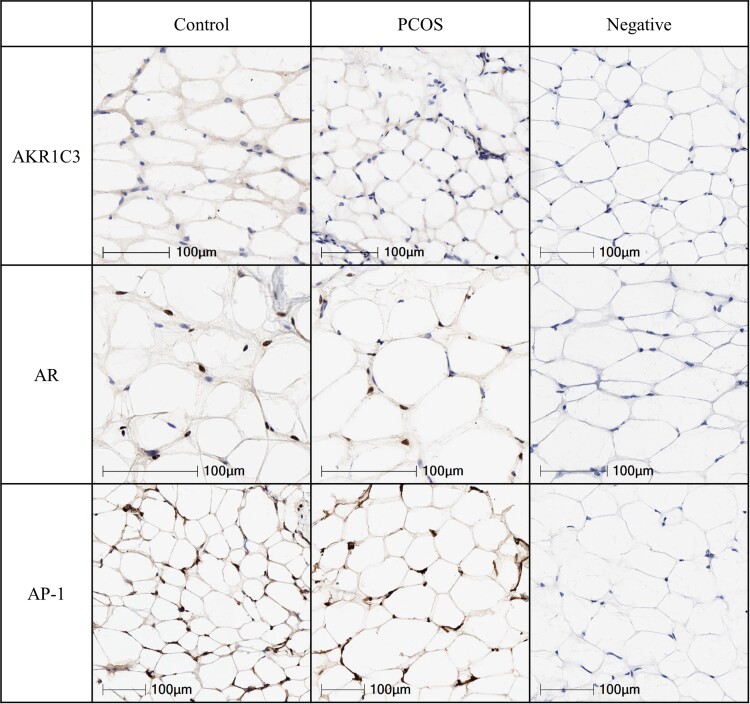
SC abdominal adipose protein expressions of AKR1C3, AR, and AP-1 in a representative normal-weight PCOS woman and age/BMI-matched control. All images were scanned at 20×, except for AR control and PCOS images, which were scanned at 30×. Abbreviations: AKR1C3, aldo-keto reductase 1C3; AP-1; activator protein-1; AR, androgen receptor; BMI, body mass index; PCOS, polycystic ovary syndrome; SC, subcutaneous.

A SC abdominal fat biopsy was performed in each woman as previously described [14], with approximately 100 milligrams of adipose tissue immediately frozen in a liquid nitrogen tank and transferred at −80 °C. Adipose tissues were sectioned to 10 to 20 micrometer sections at −20 °C and warmed up to room temperature for 30 minutes. Immunohistochemistry was performed using the Bond Polymer Refine Detection kit following standard protocol (Cat# DS9800 Leica Biosystems, Heidelberger, Germany). For detection of adipose proteins of interest, primary antibodies to AR [Cell Marque Cat# 200R, dilution 1:500, RRID:AB_2893478 (https://www.antibodyregistry.org/AB_2893478)], AKR1C3 [Abcam, Cat# ab137545, dilution 1:500, RRID:AB 3086686 (https://www.antibodyregistry.org/AB_3086686)], and AP-1/c-Jun antibody [Sigma-Aldrich, Cat # A5968, dilution 1:500, RRID:AB_258268 (https://antibodyregistry.org/AB_258268)] were used (Fig. 1).

Brightfield slides were digitized on a ScanScope AT2 (Leica Biosystems, Inc., Vista, CA) at 20 to 30× magnification. Adipocyte area was determined using Aperio Image Scope software and morphometric analysis performed with *Definiens*' Tissue Studio (Definiens Inc., Parsippany, NJ) to determine the positive index of AR protein expression (positive nuclei divided by total nuclei) and percentage of AKR1-C3 and AP-1 positive cells in a nonbiased method. Briefly, a stain-specific algorithm was created using the predefined cellular detection module and classification tool, and positive and negative stained cells within a core were identified. Thresholds were set to classify hematoxylin stain for nuclei and 3,3′-diaminobenzidine stain for positive cellular staining. The data were exported to Excel for further statistical analysis.

Scanning and analyses were performed through the Translational Pathology Core Laboratory, Department of Pathology and Laboratory Medicine, David Geffen School of Medicine at the University of California, Los Angeles.

### Statistical Analysis

**Figure 2.**
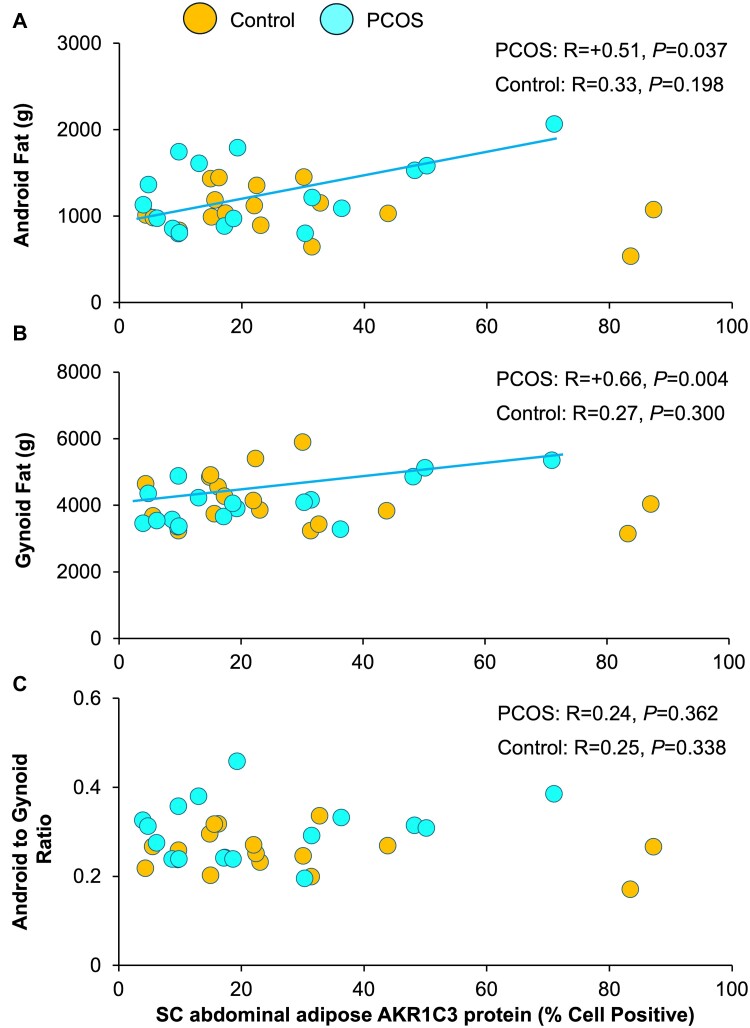
Correlations of SC abdominal adipose AKR1C3 protein expression with (a) android fat mass, (b) gynoid fat mass, and (c) android to gynoid ratio in normal-weight PCOS women and age/BMI-matched controls. Brown-colored circles, controls; teal-colored circles, PCOS women. Abbreviations: AKR1C3, aldo-keto reductase 1C3; BMI, body mass index; PCOS, polycystic ovary syndrome; SC, subcutaneous.

**Figure 3.**
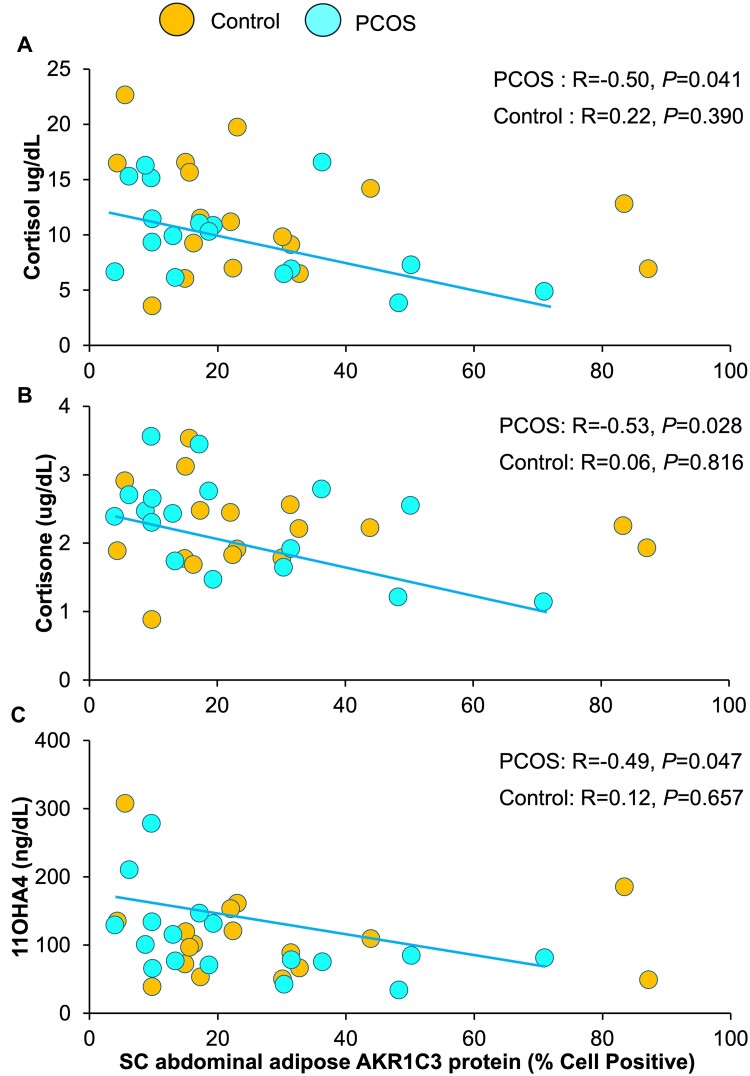
Correlations of SC abdominal adipose AKR1C3 protein expression with serum (a) cortisol, (b) cortisone, and (c) 11OHA4 levels in normal-weight PCOS women and age/BMI-matched controls. Brown-colored circles, controls; teal-colored circles, PCOS women. Abbreviations: 11OHA4, 11-hydroxyandrostenedione; AKR1C3, aldo-keto reductase 1C3; BMI, body mass index; PCOS, polycystic ovary syndrome; SC, subcutaneous.

**Figure 4.**
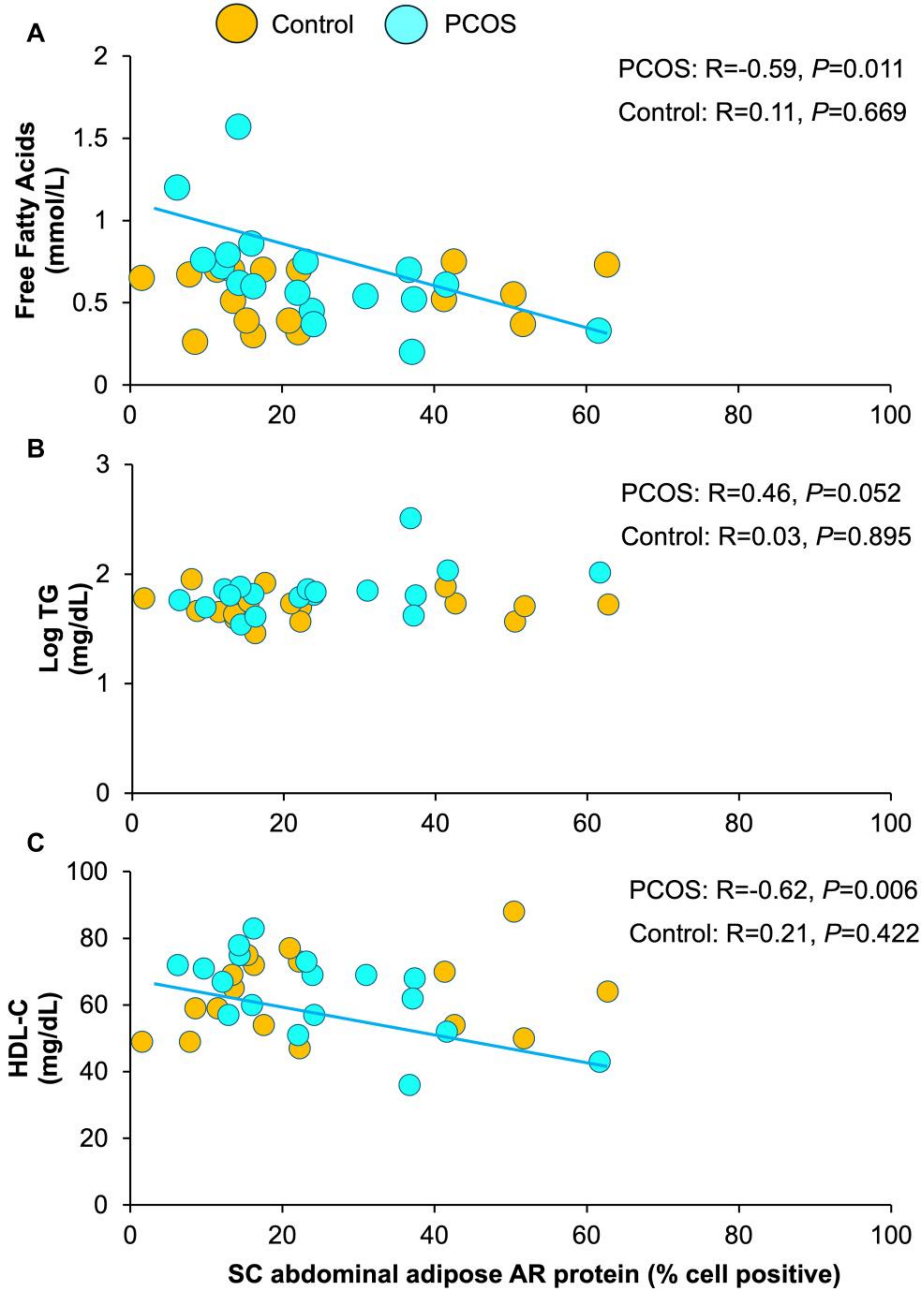
Correlations of SC abdominal adipose AR protein expression with fasting serum (a) free fatty acids, (b) log TG, and (c) HDL-C levels in normal-weight PCOS women and age/BMI-matched controls. Brown-colored circles, controls; teal-colored circles, PCOS women. Abbreviations: AR, androgen receptor; BMI, body mass index; HDL-C, high-density lipoprotein-cholesterol; PCOS, polycystic ovary syndrome; SC, subcutaneous; TG, triglyceride.

**Figure 5.**
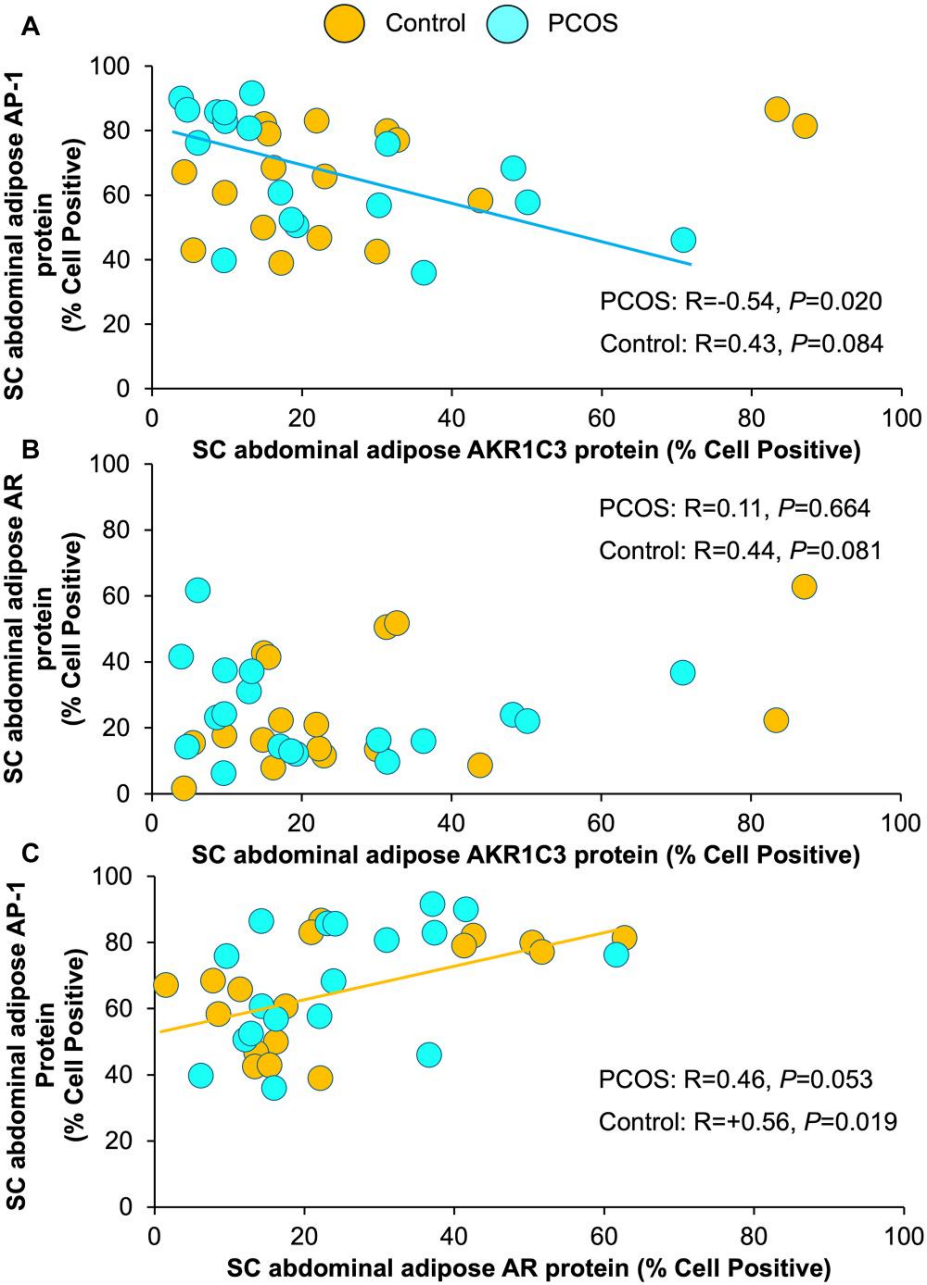
Correlations of SC abdominal adipose AKR1C3 protein expression with SC abdominal adipose (a) AP-1 and (b) AR protein expressions as well as correlations of SC abdominal adipose AR protein expression with (c) SC abdominal adipose AP-1 protein expression in normal-weight PCOS women and age/BMI-matched controls. Brown-colored circles, controls; teal-colored circles, PCOS women. Abbreviations: AP-1, activator protein-1; AR, androgen receptor; AKR1C3, aldo-keto reductase 1C3; BMI, body mass index; PCOS, polycystic ovary syndrome; SC, subcutaneous.

An unpaired Student's *t*-test compared patient characteristics, clinical hormone/metabolic values, and immunohistochemical data between PCOS and age- and BMI-matched controls. Results were presented with mean ± SD unless otherwise noted. Pearson correlation coefficients examined associations between SC abdominal adipose AKR1C3, AR, and AP-1 protein expressions as well as with clinical outcomes by study group [25]. As a sensitivity analysis, partial correlations were examined with the associations of SC abdominal adipose AKR1C3 protein expression with serum cortisone and 11OHA4 levels after adjusting for serum cortisol level to determine whether cortisol was confounding the SC abdominal adipose AKR1C3 protein findings. For significant associations, we also fit linear regression models and the prediction lines were superimposed on the scatterplots for Figs. 2, 3, 4 and 5. Due to potential distributional assumption violations (eg, nonnormality), a log transformation was applied to some measures prior to analysis (eg, LH, AIRg, TG).

In order to assess for differential correlations between study groups, we performed general linear models analyses. Model terms were the same as employed in study group specific correlations but were developed for the complete cohort, including an interaction term for study group (ie, control/PCOS). These models allowed us to test for a slope difference between study groups and to determine interaction term *P*-values. Statistical analyses were performed using IBM SPSS V27 (Armonk, NY) and *P*-values <.05 were considered statistically significant.

## Results

### Patient Characteristics, Hormone/Metabolic Levels and Immunohistological Data

As previously reported, age, BMI, and waist and hip measurements were comparable between PCOS and control women, as were total body mass, total body fat, and percent body fat. The android to gynoid fat mass ratio was significantly greater in PCOS women than controls (*P* = .027) due to a shift away from percent gynoid fat mass (*P* = .028; Table 1).

**Table 1.** Patient characteristics and serum hormone and metabolic levels in normal-weight control vs PCOS women*^a,b^*

Patient characteristics	Control (n = 17)	PCOS (n = 18)	*P*-value
Age (years)	27.5 ± 4.9	25.5 ± 4.9	.227
BMI (kg/m^2^)	21.7 ± 1.7	22.2 ± 1.9	.489
Waist (cm)	75.5 ± 5.0	76.4 ± 4.7	.579
Hip (cm)	89.6 ± 6.0	88.2 ± 5.3	.477
Total body mass (kg)	61.3 ± 7.7	61.4 ± 7.3	.953
Total body fat	19.4 ± 3.3	19.9 ± 3.2	.642
Percent body fat (%)	31.6 ± 2.9	32.6 ± 4.1	.418
Android fat (kg)	1.1 ± 0.3	1.3 ± 0.4	.138
Percent android fat (%)	5.5 ± 0.7	6.2 ± 1.3	.067
Gynoid fat (kg)	4.2 ± 0.8	4.1 ± 0.7	.714
Percent gynoid fat (%)	21.5 ± 1.6	20.5 ± 0.9	.028
Android/gynoid fat ratio	0.26 ± 0.04	0.30 ± 0.07	.027
Hormone/metabolic levels			
Log LH (mIU/mL)	0.90 ± 0.23	1.15 ± 0.22	.003
FSH (mIU/mL)	6.3 ± 2.3	5.2 ± 1.4	.092
E1 (pg/mL)	60.2 ± 30.0	72.3 ± 30.9	.250
E2 (pg/mL)	100.3 ± 108.6	75.9 ± 58.1	.419
Total T (ng/dL)*^c^*	26.2 ± 6.4	56.3 ± 20.3	<.001
Free T (pg/mL)*^c^*	3.3 ± 1.4	8.5 ± 3.2	<.001
A4 (ng/dL)*^c^*	106.4 ± 37.3	216.3 ± 75.8	<.001
11OHA4 (ng/dL)*^c^*	112.5 ± 66.0	109.6 ± 61.2	.897
DHEAS (μg/dL)	180.5 ± 103.4	215.7 ± 67.3	.247
Cortisol (μg/dL)*^c^*	11.7 ± 5.2	9.93 ± 1.0	.275
Cortisone (μg/dL)*^c^*	2.2 ± 0.6	2.3 ± 0.7	.642
Fasting glucose (mg/dL)*^d^*	85.8 ± 6.1	85.9 ± 4.5	.960
Fasting insulin (μU/mL)*^d^*	4.9 ± 2.0	6.1 ± 2.3	.111
Si (x10^−4^/min/μU/mL)*^d^*	6.7 ± 5.3	4.2 ± 2.0	.096
Log AIRg (μU/mL)*^d^*	2.4 ± 0.2	2.5 ± 0.3	.325
SHBG (nmol/L)	66.9 ± 34.9	49.6 ± 27.6	.111
Fasting FFA (mmol/L)	0.5 ± 0.2	0.7 ± 0.3	.108
Log triglyceride (mg/dL)	1.7 ± 0.1	1.8 ± 0.2	.029
HDL-C (mg/dL)	63.2 ± 11.9	63.5 ± 12.4	.937
Non-HDL-C (mg/dL)	91.5 ± 26.6	96.1 ± 26.7	.613
LDL-C (mg/dL)	80.8 ± 24.5	79.9 ± 25.3	.921
Total cholesterol (mg/dL)	155.7 ± 31.8	159.6 ± 27.0	.699
Immunohistochemical*^e^*			
AKR1C3 (% positive cells)	27.9 ± 23.9	22.3 ± 18.7	.441
AR (% positive index)	24.7 ± 18.0	24.5 ± 14.1	.968
AP-1 (% positive cells)	65.3 ± 16.3	67.9 ± 18.2	.649

Mean ± SD.

Conversion to SI units: T (X 0.0347 nmol/L), free T (X 3.47 pmol/L), A4 (X 0.0349 nmol/L), OHA4 (X 0.0331 nmol/L), DHEAS (X 0.0271 μmol/L), cortisol (X 0.0276 nmol/L), cortisone (X 0.0277 nmol/L), E1 (X 3.699 pmol/L), E2 (X 3.67 pmol/L), LH (X 1.0 IU/L), FSH (X 1.0 IU/L), glucose (X 0.0555 mmol/L), insulin (X 7.175 pmol/L, total cholesterol (X 0.0259 mmol/L), HDL-C (X 0.0259 mmol/L), LDL-C (X 0.0259 mmol/L), non-HDL-C (X 0.0259 mmol/L), triglycerides (X 0.0113 mmol/L).

Abbreviations: 11OHA4, 11-hydroxyandrostenedione; A4, androstenedione; AKR1C3, aldo-keto reductase 1C3; AP-1, activator protein-1; AR, androgen receptor; BMI, body mass index; DHEAS, dehydroepiandrosterone sulfate; E1, estrone; E2, estradiol; FFA, free fatty acids; HDL-C, high-density lipoprotein-cholesterol; LDL-C, low-density lipoprotein-cholesterol; PCOS, polycystic ovary syndrome; SC, subcutaneous; Si, sensitivity index; T, testosterone.

^*a*^Modified from references previously reported for these subjects [8-11, 14, 15].

^*b*^Total body dual-energy x-ray absorptiometry studies (Control = 17, PCOS = 17).

^*c*^Determined by liquid chromatography-tandem mass spectrometry at the University of Michigan, Ann Arbor [22, 23] (Control = 17, PCOS = 17).

^*d*^Frequently sampled intravenous glucose tolerance testing (Control = 16, PCOS = 18).

^*e*^All immunohistochemical studies were performed on subcutaneous abdominal adipose tissue.

Serum log LH, total/free T, and A4 levels were higher in PCOS women than controls (log LH, *P* = .003; androgens, *P* < .001) (Table 1). Serum 11OHA4, DHEAS, cortisol, and cortisone levels were comparable between PCOS women and controls, as were Si and log AIRg values derived from the frequently sampled intravenous glucose tolerance test. Fasting serum log TG levels were higher in PCOS women than controls (*P* = .029). There were no significant study group differences in serum levels of FSH, estrogen, SHBG or fasting glucose, insulin, FFA, and cholesterol.

New data from the present immunohistochemical analysis also demonstrated that there were no significant study group differences for SC abdominal adipose protein expressions of AKR1C3, AR, or AP-1 (Table 1, Fig. 1).

### Clinical Correlations

#### Control women

Subcutaneous abdominal adipose AKR1C3 protein expression was unrelated to total body fat or its distribution (Fig. 2A-2C, Table 2). It also was unrelated to serum levels of total/free T (total T, *P* = .444; free T, *P* = .601), cortisol (*P* = .390), cortisone (*P* = .816), 11OHA4 (*P* = .657), DHEAS (*P* = .225), or other clinical outcomes (Fig. 3A-3C, Table 2). SC abdominal adipose AR and AP-1 protein expression did not correlate with any clinical outcomes (AR: Fig. 4A-4C; AP-1: data not shown, Table 2).

**Table 2.** Differential SC abdominal adipose proteins correlations between normal-weight control vs PCOS women

Correlations	Overall	*P*-value	Controls	*P*-value	PCOS	*P*-value	Interaction
SC adipose AKR1C3 vs android fat	0.09	.607	−0.33	.198	0.51	.**037**	**0**.**010**
SC adipose AKR1C3 vs Gynoid fat	0.11	.529	−0.27	.300	0.66	.**004**	**0**.**008**
SC adipose AKR1C3 vs serum Cortisol	−0.30	.087	−0.22	.390	−0.50	.**041**	0.449
SC adipose AR vs serum FFA	−0.28	.107	0.11	.669	−0.59	.**011**	**0**.**008**
SC adipose AR vs serum HDL-C	−0.17	.317	0.21	.422	−0.62	**0**.**006**	**0**.**009**
SC adipose AKR1C3 vs SC adipose AP-1	−0.05	.781	0.43	.084	−0.54	.**020**	**0**.**003**
SC adipose AR vs SC adipose AP-1	0.50	.**002**	0.56	.**019**	0.46	.053	0.791

All values highlighted in bold are statistically significant with a *P* value < .05.

Abbreviations: AKR1C3, aldo-keto reductase 1C3; AP-1, activator protein-1; AR, androgen receptor; FFA, free fatty acids; HDL-C, high-density lipoprotein-cholesterol; PCOS, polycystic ovary syndrome; SC, subcutaneous.

#### PCOS women

In PCOS women, SC abdominal adipose AKR1C3 protein expression positively correlated with total body (R = +0.57, *P* = .017), android (R = +0.51, *P* = .037), and gynoid (R = +0.66, *P* = .004) fat masses but did not predict the android to gynoid fat mass ratio (*P* = .362) (Fig. 2A-2C, Table 2). Conversely, SC abdominal adipose AKR1C3 protein expression negatively correlated with serum cortisol (R = −0.50, *P* = .041), cortisone (R = −0.53, *P* = .028), and 11OHA4 (R = −0.49, *P* = .047) levels (Fig. 3A-3C, Table 2). Partial correlations were examined after adjusting for serum cortisol to determine whether cortisol was confounding the relationship of SC abdominal adipose AKR1C3 protein expression with serum cortisone and 11OHA4. Cortisol was chosen as a primary confounding variable for interpreting the adrenal steroidogenic data since it is the principal adrenal steroid responsible for hypothalamic-pituitary-adrenal negative feedback [26]. Adjusting for serum cortisol, SC abdominal adipose AKR1C3 protein expression was no longer related to serum cortisone (*P* = .263) or 11OHA4 levels (*P* = .583). Subcutaneous abdominal adipose AKR1C3 protein expression did not correlate with any other clinical relationship.

SC abdominal adipose AR protein expression negatively correlated with fasting FFA levels (R = −0.59, *P* = .011) (Fig. 4A, Table 2). It also positively correlated with the fasting serum log TG/HDL-C ratio (R = +0.54, *P* = .021) due to an inverse relationship with serum HDL-C levels (R = −0.62, *P* = .006) (Fig. 4B and 4C, Table 2). SC abdominal adipose AR protein expression did not correlate with other clinical outcomes.

SC abdominal adipose AP-1 protein expression negatively correlated with log serum LH levels (R = −0.52, *P* = .025).

### Associations Between Expression of SC Abdominal Adipose Proteins

#### Controls

SC abdominal adipose AKR1C3 protein expression was not significantly correlated with SC abdominal adipose AP-1 or AR protein expression (Fig. 5A and 5B, Table 2). SC abdominal adipose AR protein expression, however, positively correlated with SC abdominal adipose AP-1 protein expression (R = +0.56, *P* = .019) (Fig. 5C, Table 2).

#### PCOS

SC abdominal adipose AKR1C3 protein expression negatively correlated with SC abdominal adipose AP-1 protein expression (R = −0.54, *P* = .020, Table 2), without significant AKR1C3-AR or AR-AP-1 relationships (Fig. 5A-5C, Table 2).

#### Differential correlations between study groups

Correlation slope differences between study groups were demonstrated for SC abdominal adipose AKR1C3 protein expression association with both android fat (interaction term, *P* = .010) and gynoid fat (interaction term, *P* = .008) mass, as well as for SC abdominal adipose AR protein expression association with fasting serum FFA (interaction term, *P* = .008) and HDL-C (interaction term, *P* = .009) level (Table 2). Correlation slope differences between study groups also existed for the association of SC abdominal adipose AKR1C3 with AP-1 protein expression (interaction term, *P* = .003) (Table 2).

In contrast, slope differences between study groups were not significant for correlations of SC abdominal adipose AKR1C3 protein expression with serum cortisol level (*P* = .449) or of SC abdominal adipose AR with AP-1 protein expression (*P* = .791) (Table 2). These 2 correlations therefore were remodeled with both study groups combined. In doing so, SC abdominal adipose AR protein expression was significantly correlated with SC abdominal adipose AP-1 protein expression (*P* = .002), while SC abdominal adipose AKR1C3 protein expression tended to correlate with serum cortisol level (*P* = .087) (Table 2).

## Discussion

### SC Abdominal Adipose Protein-Clinical Associations

As a determinant of body fat deposition in humans, AKR1C enzymes located predominantly in SC adipose tissue regulate local androgen turnover [4, 6, 27]. In healthy men and women, AKR1C3 is present in SC abdominal adipose over a wide BMI range [28], with AKR1C3 mRNA expression in this adipose depot positively correlated with waist-to-hip ratio in both sexes [28], along with BMI and percent truncal fat mass independent of BMI in women [5, 6].

Approximately 25% of SC abdominal adipocytes expressed AKR1C3 protein in both normal-weight PCOS women and age/BMI-matched controls. Normal SC abdominal adipose AKR1C3 protein expression in our normal-weight PCOS women complements previous reports of enhanced SC abdominal adipose *AKR1C3* mRNA expression and activity in overweight PCOS women, implicating hyperinsulinemia from adiposity-dependent insulin resistance with SC adipose AKR1C3 activity in some, but not all, studies [7, 29]. Our data also complement findings of normal SC abdominal AKR1C3 protein levels in other PCOS women, despite elevated *AKR1C3* mRNA expression in these individuals perhaps due to protein instability, altered protein turnover, or posttranscriptional processing [29].

AKR1C3-mediated T generation in PCOS women enhances SC fat storage through increased lipogenesis and decreased lipolysis, with different metabolic responses than those of controls [7]. In agreement, SC abdominal adipose AKR1C3 protein expression in normal-weight PCOS women positively correlated with total body, android, and gynoid fat masses, rather than the android to gynoid fat mass ratio, with significantly different correlations from controls. SC abdominal AKR1C3-mediated T generation in normal-weight PCOS women compared to age/BMI-matched controls therefore predicts SC fat mass per se rather than body fat distribution within a normal BMI range, given that AKR1C3 gene expression and activity also are greater in gluteal than omental adipose, with gluteal adipose favoring androgen activation [6].

SC abdominal adipose AKR1C3 protein expression in normal-weight PCOS women correlated negatively with serum cortisol, cortisone, and 11OHA4 levels. Moreover, serum cortisone and 11-OHA4 levels were no longer significant after adjusting for serum cortisol as the principal steroid responsible for hypothalamic-pituitary-adrenal negative feedback regulation [26]. These inverse relationships between SC abdominal adipose AKR1C3 protein expression and serum cortisone and 11OHA4 levels, therefore, could be influenced by cortisol, although the exact nature of these complex relationships may also be affected by other factors that will require further investigation [30]. This inverse relationship between SC abdominal adipose AKR1C3 protein expression and circulating cortisol levels was not statistically different between women with PCOS and controls, implying that SC fat storage in normal-weight women may represent a reciprocal balance between androgen inhibition of catecholamine-induced lipolysis [31, 32], as seen in similar adipose of PCOS women [33, 34], and peripheral glucocorticoid-induced lipolysis [26, 35-38]. If so, given cortisol as an insulin antagonist, diminished cortisol activity relative to androgen excess in normal-weight PCOS women could favor glucose uptake in target tissues, together with reduced breakdown of muscle proteins and TGs, to simultaneously promote energy use in combination with lipid storage and muscle strength in the presence of increased intra-abdominal fat mass [8, 14, 26, 38].

Subcutaneous abdominal adipose AR protein expression also was similar between normal-weight PCOS women and age/BMI-matched controls. A negative correlation of SC abdominal adipose AR protein expression with fasting serum FFA levels in women with PCOS, however, differed from that of controls, perhaps due to enhanced SC fat storage, limiting fatty acid substrate for hepatic glucose production [39]. Similarly, SC abdominal adipose AR protein expression in PCOS women positively correlated with fasting serum log TG/HDL-C ratios due to an inverse relationship with serum HDL-C levels, in contrast to the absence of comparable correlations in controls, implying additional AR-mediated hepatic actions through increased intra-abdominal fat mass [14, 27, 32, 40-42]. In support of this, dual brain-adipocyte AR knock-out, but not brain-specific AR knock-out, in a PCOS-like mouse model completely protects against both hepatic steatosis and parametrial adipocyte hypertrophy [43, 44].

### Associations Between Expression of SC Abdominal Adipose Proteins

The positive correlation between SC abdominal adipose AR and AP-1 protein expression in both study groups combined links AR activation with AP-1 activity, perhaps through Yes-associated protein/transcriptional coactivator with PDZ-binding motif (TAZ)-SV40 transcriptional enhancer factor domain transcriptional complex, as seen in prostate cancer cell lines [45-48]. This transcriptional complex modulates adipocyte proliferation vs differentiation via Hippo signaling, and in association with AR/AP-1 through TAZ, could mediate SC adipose development [49, 50]. Alternatively, AR/AP-1 in association with the MAPK/c-Jun N terminal kinase pathway [51] also could promote SC abdominal adipogenesis [52].

In contrast, a negative correlation of SC abdominal adipose AKR1C3 and AP-1 protein expression in PCOS women differed from that of controls, perhaps linking AP-1 or TAZ with the glucocorticoid receptor [53, 54] and AR/AP-1 transcriptional interference [55-59]. In normal-weight PCOS women, therefore, an association between SC abdominal adipose AKR1C3-mediated T generation and circulating cortisol could repress AP-1 transcription to counterbalance enhanced developmentally programmed AP-1 activity during accelerated adipogenesis in this adipose depot [9, 11, 12, 35, 60]. Such intra-adipose events would be further supported by hCG/LH inhibition of AP-1 activation as seen in other cell lines [61, 62], agreeing with the presence of hCG/LH receptors in human SC adipose [63] and the negative correlation of serum log LH levels with SC abdominal adipose AP-1 protein expression in our women with PCOS but not in our controls.

Important strengths of this study were the use of healthy, normal-weight PCOS women by NIH criteria who were age- and BMI-matched to controls to eliminate confounding effects of age and obesity [64, 65]. Our experimental design also reduced the confounding effects of referral bias on metabolic outcomes [66, 67]. In addition, fasting blood samples were collected at 10 Am to avoid diurnal variation of adrenal steroidogenesis, allowing us to study the relationship of SC abdominal adipose AKR1C3 protein expression with serum cortisol levels. Despite these study strengths, however, our study was not a population-based study and also was unable to completely eliminate selection bias given its exclusive use of volunteers or consider subject variability in the diurnal timing or peak production of adrenal steroidogenesis.

As additional limitations of our study, we did not investigate the expression or activity of other steroid aldoketoreductases 1C by adipose depot, as others have done [68], nor did we examine adipose steroid metabolism, which could have influenced associations examined. Furthermore, power limitations may have limited our ability to detect a reduced serum cortisol to cortisone ratio, as a biomarker of decreased HSD11ß1 activity, as previously reported in a larger number of these normal-weight PCOS women and others [8, 69]. The small number of PCOS subjects also diminished statistical power to examine subtle female-type differences in SC abdominal adipose protein expressions and limited applicability of our findings to women of different PCOS phenotypes, ethnicity, or adiposity. Finally, our study did not investigate the effects of glucocorticoid receptor protein expression or activity in adipose on energy storage or AP-1 transcription [12, 35, 55, 70-73] as potential PCOS-related links with adiposity and inflammation [74, 75].

Nevertheless, our findings link SC abdominal adipose AKR1C3 protein expression with metabolic adaptations of normal-weight PCOS women [76-79]. In normal-weight PCOS women, SC abdominal adipose AKR1C3 protein expression in combination with adipocyte differentiation, developmentally programmed by AP-1 transcription factors and AR-dependent events, predicts fat accretion in the presence of physiological cortisol levels. Such a metabolic adaptation originally regulated energy homeostasis and body fat development to ensure survival and reproduction but now predisposes women with PCOS to excess weight gain and lipotoxicity in today's obesogenic environment [76-80].

## Data Availability

Some or all datasets generated during and/or analyzed during the current study are not publicly available but are available from the corresponding author on reasonable request.
